# Nanoscale electrochemical response of lithium-ion cathodes: a combined study using C-AFM and SIMS

**DOI:** 10.3762/bjnano.9.154

**Published:** 2018-06-04

**Authors:** Jonathan Op de Beeck, Nouha Labyedh, Alfonso Sepúlveda, Valentina Spampinato, Alexis Franquet, Thierry Conard, Philippe M Vereecken, Wilfried Vandervorst, Umberto Celano

**Affiliations:** 1 IMEC, Kapeldreef 75, 3001 Leuven, Belgium; 2 KU Leuven, Department of Physics and Astronomy, Celestijnenlaan 200D, B-3001 Leuven, Belgium; 3 KU Leuven, Department of Microbial and Molecular Systems, Celestijnenlaan 200D, B-3001 Leuven, Belgium

**Keywords:** all-solid-state microbatteries (ASB), conductive atomic force microscopy (C-AFM), Li-ion kinetics, secondary ion mass spectrometry (SIMS), 3D thin-film batteries

## Abstract

The continuous demand for improved performance in energy storage is driving the evolution of Li-ion battery technology toward emerging battery architectures such as 3D all-solid-state microbatteries (ASB). Being based on solid-state ionic processes in thin films, these new energy storage devices require adequate materials analysis techniques to study ionic and electronic phenomena. This is key to facilitate their commercial introduction. For example, in the case of cathode materials, structural, electrical and chemical information must be probed at the nanoscale and in the same area, to identify the ionic processes occurring inside each individual layer and understand the impact on the entire battery cell. In this work, we pursue this objective by using two well established nanoscale analysis techniques namely conductive atomic force microscopy (C-AFM) and secondary ion mass spectrometry (SIMS). We present a platform to study Li-ion composites with nanometer resolution that allows one to sense a multitude of key characteristics including structural, electrical and chemical information. First, we demonstrate the capability of a biased AFM tip to perform field-induced ionic migration in thin (cathode) films and its diagnosis through the observation of the local resistance change. The latter is ascribed to the internal rearrangement of Li-ions under the effect of a strong and localized electric field. Second, the combination of C-AFM and SIMS is used to correlate electrical conductivity and local chemistry in different cathodes for application in ASB. Finally, a promising starting point towards quantitative electrochemical information starting from C-AFM is indicated.

## Findings

Conventional Li-ion battery technology is undergoing continuous improvements in order to fulfil the increasing demands from modern society on autonomous electronics, such as portable devices, internet-of-things applications and implants [1]. A multitude of studies have already indicated that nanotechnology, nanostructured designs and nanocomposite materials will play an important role for future Li-ion batteries [1–3]. The 3D all-solid-state microbattery (ASB) is a promising new architecture built using processing techniques compatible with semiconductor processing, which provides more power and more capacity compared to conventional planar designs [4]. In this kind of battery, the electrolyte is generally a solid and dense material while crystalline conductive oxides are used for the anode and cathode. As a solid electrolyte is significantly safer compared to its flammable organic liquid counterparts, its use does represent a clear advantage [2]. Moreover, the presence of crystalline ordering in the anode and cathode, creates high-mobility channels for the lithium migration, thus significantly enhancing the ionic conductivity of these materials [5].

However, being based on diffusion in a solid versus a liquid, the success of ASB will depend on the capability to address the nanoscale ionic processes in the thin films, at their interfaces and the combined electronic–ionic transport. It goes without saying that in sub-micrometer films thickness, the nanoionic properties of the system become more dominant and, similarly, the interfaces between layers represent a higher (compared to bulk) volume fraction in the final cell. This represents a criticality for virtually all battery technologies. However, as we focus on ASB technology the solid–solid interaction creates new challenges due to the different nature of the established interfaces. Uncontrolled side reactions and phase decomposition between the electrodes and the electrolyte, which are complex to characterize, give rise to failure and reduced performance of cells. This puts the outcome of our work in context of a wide range of applications. These pending problems pose severe challenges for the physical characterization of battery materials such as the local correlation between a nanoscale stimulus and the resulting dynamically evolving material response [6].

In this letter, we propose a solution to study the nanoscale characteristics of ASB materials by a combinatorial approach that uses two established analysis techniques such as conductive atomic force microscopy (C-AFM) and secondary ion mass spectrometry (SIMS). As model systems, we focus on LiMn_2_O_4_ (LMO) as cathode material [7] deposited by wet electrodeposition (thickness 260 nm) and RF-sputtered (thickness 100 nm) and compare their properties on a local (sub-100 nm) scale. In addition, a comparison is made with pristine electrodeposited MnO_2_ (thickness roughly 250 nm) before conversion to LMO by solid-state reaction; this is done to have a reference sample that does not contain lithium.

**Figure 1:**
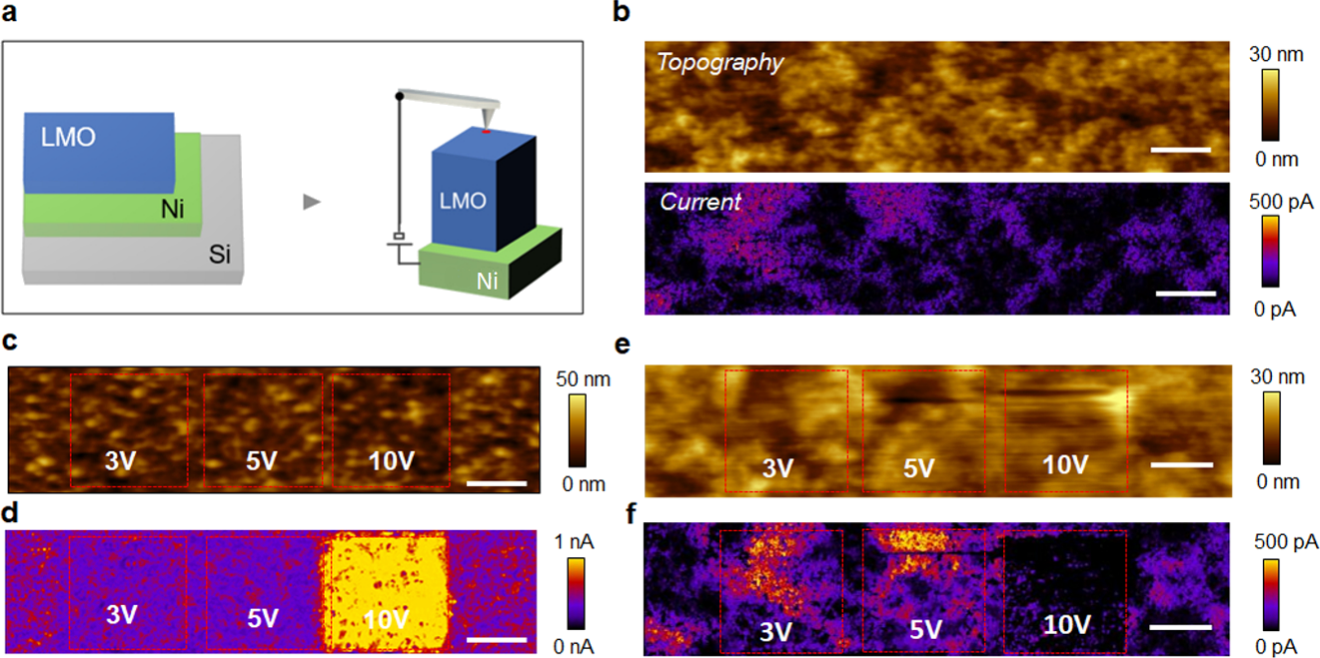
C-AFM configuration and study of the influence of an applied voltage stress on MnO_2_ and LMO. (a) Schematic of the C-AFM setup and sample structure. The tip is grounded while the dc bias is applied to the sample. (b) Topography and current maps as collected by C-AFM on the electrodeposited LMO sample applying 1.5 V. (c) MnO_2_ morphology and (d) current map reporting three areas previously stressed at different dc bias values applying 1.5 V. (e) Electrodeposited LMO morphology and (f) current map reporting three areas previously stressed at different dc bias values applying 1.5 V (scale bar 1 µm for all images).

The general structure of our samples and the C-AFM setup are schematically shown in Figure 1a. The three samples mentioned above are all deposited on a metallic current collector (Ni or Pt) on top of a silicon wafer. Spatially resolved electrical properties are observed with nanometer resolution by scanning a biased conductive AFM tip across the top surface. Unless specified otherwise, we apply always the bias to the sample (i.e., the metallic Ni/Pt layer) while the C-AFM tip is grounded. By measuring the current (using the tip as a nanoscale electrode) and the tip deflection as a function of the AFM tip position, two-dimensional maps of the local conductivity and the topography can be formed. For instance, Figure 1b shows the topography and current distribution map when performing such measurements in the case of the electrodeposited LMO. This basic concept was extended with the development of various scanning probe microscopy (SPM) techniques [8–12] dedicated to probe ionic dynamics such as the observation of motion of ions in mixed ionic–electronic conductors using the electrical current sensed by the AFM tip [6].

However, these techniques all probe the electrical, structural or ionic properties of the film and do not provide any direct information on the local chemistry, which is an important (missing) piece of information. For this reason, inspired by the work of E. Strelcov et al. [10] on the first-order reversal curve current–voltage (FORC-IV) method on ferroelectrics, we will investigate the fundamentals behind tip-induced sensing in Li-ion cathodes using a hybrid metrology approach combining SPM with SIMS, this is a technique able to observe the actual Li concentration.

Using the C-AFM tip as a nanoscaled electrode, we can now stress films at different bias values by scanning over the surface (8.5 min) and subsequently observe the induced conductivity changes. In Figure 1c–f we show the impact of different (tip-induced) voltage stresses applied under ambient conditions on two electrodeposited cathodes, i.e., MnO_2_ before (Figure 1c,d) and after lithium insertion (LMO, Figure 1e,f). From the resulting modifications of the current maps (Figure 1d,f) it is clear that both films behave very differently. As visible in Figure 1d, MnO_2_ shows no significant changes in conductivity after stressing with a positive sample bias of 3 V and 5 V. On the contrary, LMO shows a strong increase in the conductivity after the application of 3 V and 5 V relative to the map for its pristine state (Figure 1b). The different conductivity changes between MnO_2_ and LMO can be attributed to the presence of lithium in LMO as this can migrate and locally accumulate at the surface driven by the applied electric field. The impact of the 10 V stress is described later in the text, as it involves a dedicated interpretation due to the relatively high voltage involved. A more quantitative comparison of the regions is shown in Figure S1 (Supporting Information File 1). The line graph indicates clearly the increasing conductivity change (69%, 160%) with increasing stress (3 V, 5 V). Worth noting is that also regions outside of our intended stress have slightly changed in local conductivity due to the readout bias (1.5 V).

We also observed that the conductivity in the layer is not entirely uniform after lithium insertion, indicating that the LMO is not achieving maximum cathode utilization. The latter could be representative of the known fact that lithium shows a strong tendency to be localized and often is trapped at grain boundaries [13]. However, while C-AFM proves to be very useful to sense the electrical properties of the cathode materials, including the areal distribution and density of highly conductive paths, it does not provide any information about the chemical composition of such regions. For this purpose, the combination C-AFM with SIMS is needed.

It is important to mention that when working in air, a nanosized electrolytic half-cell is formed at the tip–sample system due to the presence of a water meniscus on all the surfaces. As shown elsewhere, this water layer can act as a Li-ion reservoir and in combination with an applied electric field at the AFM tip it can induce multiple oxidation processes leading to the formation of insulating Li-compounds (e.g., Li_2_O and Li_2_CO_3_) [14]. This has a large influence on the C-AFM measurement as the insulating compounds on the top surface impact on the observed conductivity, up to preventing all electrical contact to the underlying film. We believe such oxide formation can also be observed in the present work since after stressing at 10 V, a net drop in conductivity (or observed current) is visible (10 V box Figure 1f). Since the observed morphology hardly changes (only a small effect is visible in case of a 10 V bias), taking tip wear into account, we believe that our bias stress up to 5 V does not induce modifications to the surface. These undesired side reactions between the tip and sample can be drastically reduced by performing the measurements under ultra-high vacuum conditions instead of normal ambient air. In the case of the 10 V bias stress applied to the MnO_2_, a drastic increase of the conductivity is observed, which can be attributed to the dielectric breakdown of the film. High vacuum condition can be achieved (see below in Figure 3a) by working with a dedicated tool combining AFM and time-of-flight SIMS (TOF-SIMS) in the same apparatus (10^−6^ mbar or lower, TOF-SIMS V, ION-TOF GmbH, Münster, Germany). The advantage of this concept is that it allows one to perform electrical analysis (using C-AFM) and chemical analysis (using TOF-SIMS) on exactly the same area with C-AFM offering a much higher spatial resolution (ca. 3–5 nm) than TOF-SIMS (50–100 nm).

**Figure 2:**
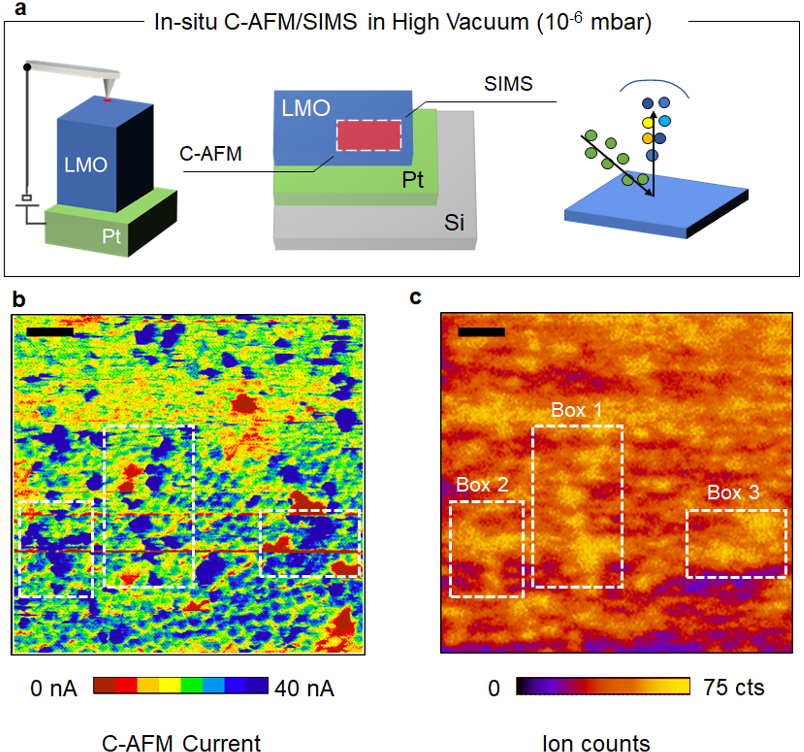
Combined C-AFM and SIMS analysis of a RF-sputtered LMO film. (a) Schematic of the measurement setup. The system is in high vacuum (10^−6^ mbar). (b) Local current map as probed by C-AFM (sample bias −8 V) and corresponding (c) local chemical profile of lithium as measured by SIMS on the same area (scale bar 2 µm). Both C-AFM and SIMS indicate the segregation of the film in phases with different local chemistry and electrical conduction. Additionally, their strong correlation reveals the inherent link between the varying Li content and electrical conductivity as highly lithiated regions show enhanced electrical conductivity.

The schematic of the experiment to perform TOF-SIMS (detecting the Li concentration at the surface) and C-AFM (local conductivity) in succession in the same area for the case of the RF-sputtered LMO cathode is shown in Figure 2a. The static SIMS analysis is performed using a Bi_3_ cluster beam (spot size ca. 70 nm) in the area previously studied by C-AFM. Static SIMS is known to be probing only the outermost monolayer with minimal disturbance ensuring the information we obtain is only the surface chemical composition [15]. In essence, the SIMS results provide the 2D distribution map of the Li concentration at the surface with a clear distinction between Li-rich and Li-deficient areas. A striking correspondence (for example in Figure 2a, boxes 1–3) between the highly conductive regions and the high Li-concentration regions can be observed indicating the relation between electrical properties the chemical composition of that area. In other words, our results show that highly lithiated domains are localized within sub-micrometer clusters, which also represent a low-resistance path for the electronic current in the cathode layer. It is worth noting that besides the regions with a clear electrical and chemical correlation, conductive spots without high Li concentration are also visible. In addition, (partial) relaxation after bias stress can lead to modifications of the surface deviating from the original C-AFM image. Figure 2 demonstrates that with our method we can distinguish two extreme material phases and their respective electrical properties. This can be interesting in studying uncomplete cathode utilization, which is often considered as one of the main bottlenecks for ASB cathodes [2,4]. For example, for the system presented in our work, we show that the material is not yet fully optimised for maximal cathode utilization, as on a local scale, the LMO shows strong variations in Li concentration and conductivity (Figure 1 and Figure 2).

**Figure 3:**
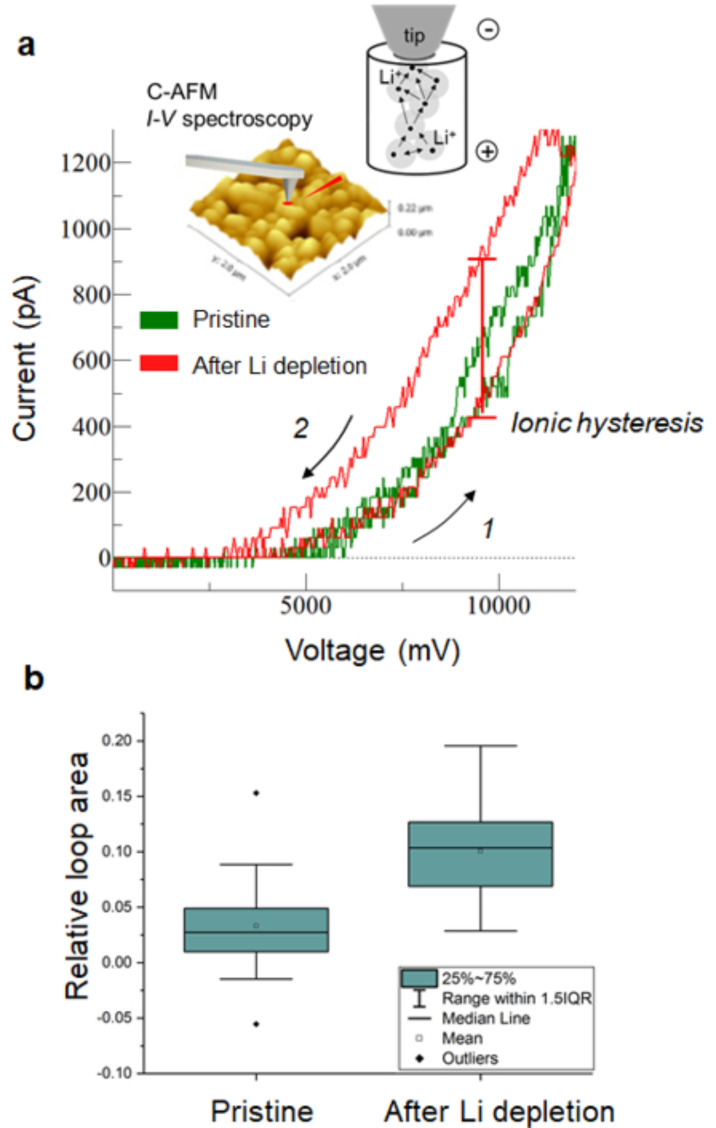
Appearance of the ionic hysteresis and influence of Li depletion during preconditioning. (a) The hysteresis loop visible in the *I*–*V* curves is indicating the local change in resistance under the tip induced by the Li modulation. The inset shows a schematic of the migration of Li ions towards the AFM tip. The measurements were performed on RF-sputtered LMO under high vacuum at a sweep rate of 0.2 Hz. (b) An enhanced hysteresis loop opening, i.e., electrochemical response, can be observed due to a preconditioning dc scan (10 V) performed to deplete the lithium before the *I*–*V* curves are measured. The boxplot shows the distribution of the relative loop area (area enclosed by the loop and divided by the peak current magnitude).

C-AFM does not allow any differentiation between the ionic and the electronic current contribution in the current map, but rather it shows the sum of both. Therefore, in this section, we discuss an approach to separate both components. Figure 1e,f proves that we can induce a local change in the conductivity of the layer when the tip is scanned with a negative polarity, which we relate to the presence of mobile Li ions in the LMO film. This effect, which has been already shown for other mixed ionic–electronic conductors, represents an interesting starting point to obtain local electrochemical information from the sample using C-AFM [6]. Indeed, the biased tip induces a strong electric field (localized under the tip) inside the material, thus triggering a field-induced ionic migration of the Li ions which, if mobile, start to accumulate at the tip–sample interface. As shown in Figure 2, such local accumulation of Li locally induces a non-volatile change in resistance under the tip. While this effect is visible in Figure 1 for the (squared) regions we show the effect in a local point-contact *I*–*V* spectroscopy measurement in Figure 3. Here the tip is held fixed in contact with the sample while the dc bias is applied to the sample (inset Figure 3a). A decrease in the local tip–sample resistance resulting from applying a bias is visible from the hysteresis in the *I*–*V* curves, which is consistently observed between the trace (1) and retrace (2) dc bias sweeps. The amplitude of the hysteresis depends on the local concentration and mobility of Li under the tip. Figure 3a shows the example of two *I*–*V* curves acquired (1) in a pristine area (green trace) and (2) in a region that was previously scanned with the tip positively biased at 10 V (red trace). The large hysteresis observed in the second case, demonstrates that by stressing the sample surface with a negative dc sample bias a strong depletion of Li ions occurs such that a higher resistance change can be obtained under the tip while attracting the Li ions back during the following positive sample bias sweep. This effect is qualitatively shown in Figure 3a. We compare a more relevant statistical population in Figure 3b, where the comparison is done evaluating 145 *I*–*V* spectroscopy curves in which the negative dc sample bias stress was performed before every single measurement for roughly half of the data set. These results indicate that although the approach distorts the initial state of the material and the Li-ion distribution, it can be an effective way of obtaining a higher response for the same applied bias, thus improving the sensitivity of a potential ion-modulated C-AFM measurement. This can be particularly useful for fundamental studies on the role of materials, grain boundaries and interfaces that provide a low bias induced response.

It is important to consider the formation of a thin local oxidized interface at the tip–sample junction as another possible origin of modified transport characteristics. Especially in air, local anodic oxidation is well known to limit C-AFM capabilities and complicate the results interpretation in the case of silicon [16]. For this reason, UHV conditions have been investigated in order to increase the reproducibility and quantitative interpretation of C-AFM [17]. At the same time, we do not expect the high-vacuum environment to be completely free from humidity. Therefore, in these measurements the formation of any Li composites such as Li_2_O and Li_2_CO_3_ cannot be excluded as also considered by others [14]. However, a local oxidation effect is a non-reversible process, while in our observation the reversibility of the conductive changes (visible in Figure 3a red curve) proves that the local formation of these compounds (although not excluded) is not the main cause for the observation.

More work is required in particular for a quantitative interpretation of the information contained in the ionic hysteresis (Figure 3a), especially the conversion of the *I*–*V* resistance shift in relevant ionic parameters, e.g., local ionic mobility or diffusivity. This is an ongoing activity and represents the topic of state-of-the-art studies [18]. However, the observations reported in Figure 3a,b combined with the results of C-AFM and SIMS of Figure 1 and Figure 2 clearly represent an important starting point toward the development of a C-AFM/SIMS-based analysis framework for battery materials.

In summary, using MnO_2_ and LMO as cathode model systems for ASB, we have demonstrated the use of combined scanning probe and beam analysis techniques to investigate electrical, structural and electrochemical properties at the nanoscale. C-AFM was used for comparing the local electrical conductivity of the two materials and shows the impact of the Li incorporation on the layer resistance. Second, by alternating on the same area C-AFM and SIMS, a direct correlation between the presence of nanosized conductive paths and Li concentration could be established. Moreover, we show the capability of a biased AFM tip to locally accumulate and deplete Li ions on/below the surface thus representing an interesting starting point towards the C-AFM-based analysis of electrochemical properties in mixed ionic–electronic conductors. These concepts can be extended to the other constituents of Li-ion batteries, such as the anode and the solid electrolyte, which share the same open issues for their nanoscale physical characterization. All-solid-state lithium batteries are considered as promising energy storage devices to meet the requirements of a low-carbon society, therefore the development of a dedicated material metrology platform is key to unlock the potentials of ASB.

## Supporting Information

File 1 Additional experimental data.
