# Supplementary material for: Nanoscale electrochemical response of lithium-ion cathodes: a combined study using C-AFM and SIMS
Source: Beilstein J Nanotechnol. 2018 Jun 4;9:1623–8. doi: 10.3762/bjnano.9.154 (PMC6009439; doi:10.3762/bjnano.9.154)
Supplement: File 1 — Additional experimental data. [file Beilstein_J_Nanotechnol-09-1623-s001.pdf]

Supporting Information

for

Nanoscale electrochemical response of lithium-ion cathodes: a combined study using C-AFM and SIMS

Jonathan Op de Beeck*[1,2], Nouha Labyedh[1,3], Alfonso Sepúlveda[1], Valentina Spampinato[1], Alexis Franquet[1], Thierry Conard[1], Philippe M. Vereecken[1,3], Wilfried Vandervorst[1,2] and Umberto Celano*[1]

Address: [1]IMEC, Kapeldreef 75, 3001 Leuven, Belgium; [2]KU Leuven, Department of Physics and Astronomy, Celestijnenlaan 200D, B-3001 Leuven, Belgium and [3]KU Leuven, Department of Microbial and Molecular Systems, Celestijnenlaan 200D, B-3001 Leuven, Belgium

Email: Jonathan Op de Beeck* - jonathan.opdebeeck@imec.be; Umberto Celano* - celano@imec.be

*Corresponding author

Additional experimental data

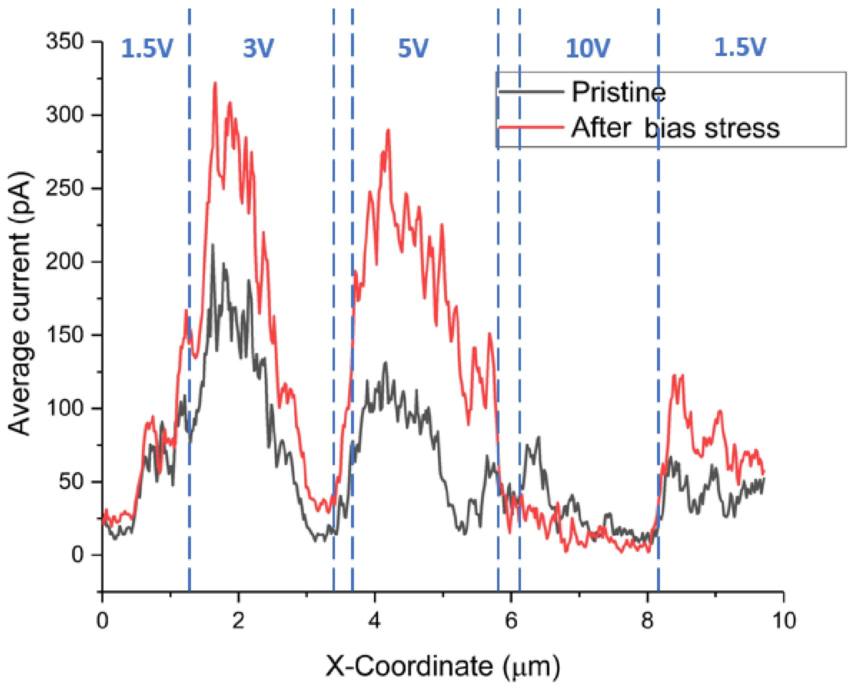

Figure S1: One-dimensional current profile (averaged over width $y \approx 1.5$ μm) showing the influence of multiple bias stress on electrodeposited LMO (cf. Figure 1b,f of the main manuscript).
